# Accelerated Sarcopenia Phenotype in the DJ-1/*Park7*-Knockout Zebrafish

**DOI:** 10.3390/antiox13121509

**Published:** 2024-12-11

**Authors:** Kristine O. Rostad, Tobias Trognitz, Ann Kristin Frøyset, Ersilia Bifulco, Kari E. Fladmark

**Affiliations:** Department of Biological Sciences, University of Bergen, 5020 Bergen, Norwayttr041@uib.no (T.T.); ann.froyset@uib.no (A.K.F.); ersilia.bifulco@uib.no (E.B.)

**Keywords:** DJ-1, park7, Parkinson’s disease, sarcopenia, skeletal muscle, mitochondria

## Abstract

Age-dependent loss of muscle mass and function is associated with oxidative stress. DJ-1/*Park7* acts as an antioxidant through multiple signalling pathways. DJ-1-knockout zebrafish show a decline in swimming performance and loss of weight gain between 6 and 9 months of age. Here, we address the degree to which this is associated with muscle degeneration and identify molecular changes preceding dysregulation of muscle performance. Loss of DJ-1 reduced the skeletal muscle fibre cross-section area. The highly mitochondrial-dependent red slow muscle was more affected than the white muscle, and degeneration of sub-sarcolemma red muscle mitochondria was observed. Using TandemMassTag-based quantitative proteomics, we identified a total of 3721 proteins in the multiplex sample of 4 and 12-month-old muscles. A total of 68 proteins, mainly associated with inflammation and mitochondrial function, were dysregulated in the young DJ-1-null adults, with Annexin A3, Sphingomyelin phosphodiesterase acid-like 3B, Complement C3a, and 2,4-dienoyl CoA reductase 1 being the most affected. The loss of DJ-1 also accelerated molecular features associated with sarcopenia, such as a decrease in the NAD^+^/NADH ratio and a reduction in Prostaglandin reductase 2 and Cytosolic glycerol-3-phosphate dehydrogenase levels. In view of the experimental power of zebrafish, the DJ-1-null zebrafish makes a valuable model for understanding the connection between oxidative stress and age-dependent muscle loss and function.

## 1. Introduction

Progressive loss of skeletal muscle is associated with various conditions, including ageing, metabolic, and neurodegenerative diseases. Increasing evidence shows that oxidative stress and inflammation may be driving factors in muscle atrophy, although in a complex relationship with other mechanisms such as endocrine factors, metabolic changes, and neuromuscular impairment [1].

Mutations in the *park7* gene encoding DJ-1 may lead to early onset Parkinson’s disease (PD). In addition, increased expression of oxidated non-functioning DJ-1 is associated with oxidative stress-related diseases such as spontaneous PD, Alzheimer’s disease, and Amyotrophic lateral sclerosis [2]. DJ-1 is a multifunctional and ubiquitously expressed protein. It is highly recognised as a regulator of oxidative stress through pro-survival pathways, transcriptional control of antioxidant genes, and mitochondrial homeostasis.

DJ-1 has an important role in regulating oxidative stress in the skeletal muscle [3]. In the absence of DJ-1, there is a shift towards glycolysis to reduce mitochondrial reactive oxygen species (ROS) accumulation in the skeletal muscle [3]. Skeletal-muscle-specific knockout of DJ-1 in mice results in reduced muscle mass and impaired mitochondrial function [4].

Vertebrate knockout models of DJ-1/*park7* have been established in mice [5,6], rats [7,8], and zebrafish [9,10]. Knockout of DJ-1 in rats and zebrafish affects dopaminergic neurons, whilst in the mice models, nigrostrial function is retained. There is also a discrepancy between mice and rat models as to whether motor function is lost or not [5,6,7,8].

Our DJ-1/*park7*-knockout zebrafish exhibits progressive, age-dependent PD-associated symptoms and pathology. Embryos seem to develop normally, but a retinal degenerating phenotype is observed in juveniles, followed by a dysregulation in the brain proteome in young adults, which precedes loss of dopaminergic neurons and altered behaviour [9,11]. Between 6 and 9 months of age, swimming distance is reduced, and anxiety-related behaviour is increased in knockout animals compared to wild-types [12,13]. Wild-type animals continue to gain weight with age, whilst knockouts have reduced weight gain from 6 months of age [12]. At 12 months of age, a marked reduction in mitochondrial Complex I activity is observed in the muscle of knockout zebrafish [9].

Zebrafish have emerged as powerful models for studying human diseases due to their small size, rapid development, ease of genetic modification, and the high similarity of their genetics and physiology to humans. Zebrafish skeletal muscle resembles human muscle in both morphology and molecular aspects [14]. Features of sarcopenia, the ageing of skeletal muscle, have been observed in [9] adult stage zebrafish (30 months stage), including loss of muscle mass and mitochondrial degradation [15]. In this respect, it is of interest to evaluate the effect of DJ-1 knockout in association with both molecular markers and features of sarcopenia.

In contrast to murine models, zebrafish has a distinct separation between highly mitochondrial-dependent slow-red and anaerobic/glycolytic fast-white muscle fibres. This feature may add to the advantage of using zebrafish to study the effect on mitochondrial function in skeletal muscle maintenance. Here, we used our previously established DJ-1-deficient zebrafish model to further elucidate the role of DJ-1 in the skeletal muscle. Morphological, metabolic, and quantitative proteomic analyses were performed to identify molecular changes that precede altered motor behaviour.

## 2. Materials and Methods

### 2.1. Zebrafish Maintenance and Lines

Zebrafish were housed at the Zebrafish Facility at the University of Bergen’s Department of Biological Sciences, which follows the European Convention for the Protection of Vertebrate Animals used for Experimental and Other Scientific Purposes. The adult zebrafish were kept at a temperature range of 26–28 °C and fed twice daily (arthemia and Zebrafeed 400–600 μm from Sparos, Olhao, Portugal) while following a 14/10 h light cycle. Conductivity and pH ranges were 500–800 and 7.2–7.6, respectively.

A *park7* knockout (DJ-1 KO) line has previously been established using the CRISPR-Cas9 method targeting exon 1 of *park7* in the Tübingen AB wild-type (WT) zebrafish line [9]. Approval for the line was granted by the National Animal Research Authority, Mattilsynet (FOTS ID8039 and ID14039).

### 2.2. Evaluation of Skeletal Muscle Cross-Sectional Area

Muscle samples from two-year-old male DJ-1 KO and WT fish were fixed with paraformaldehyde diluted to 4% in 1× PBS at 4 °C. After fixation, the samples were decalcified for 3 h in buffered formic acid (0.5 M NaOH, 22.5% CH_2_O_2_), followed by washing in 1× PBS and dehydration with 70% and 96% ethanol for 15 min. The samples were embedded in Technovit 7100 (Heraeus Kulzer GmbH, Hanau, Germany) and left to solidify for two days. The samples were cut into 2 µm thin sections using a microtome (Leica RM 2165) and stained with Toluidine blue (2% (*w*/*v*) Borax, 1% (*w*/*v*) Toluidine blue). Images were taken with a slide scanner (Zeiss, AxioScan.Z1, Oberkochen, Germany). The cross-sectional area and number of myofibers were measured and counted manually in Fiji (ImageJ (https://imagej.net/ij/)) from three animals from each group. GraphPad Prism 10.0 was used for statistical analysis.

### 2.3. Ultrastructural Analysis

Muscle samples from 18-month-old male DJ-1 KO and WT fish were fixed with 2.5% glutaraldehyde and 2% paraformaldehyde in 0.1 M sodium cacodylate buffer for 48 hrs. Samples were then washed with 0.1 M sodium cacodylate buffer and stored at 4 °C until use. The fixed muscle samples were post-fixed with 1% osmium tetroxide in 0.1 M sodium cacodylate buffer, followed by washing in 0.1 M sodium cacodylate buffer and dehydrating with increasing ethanol solutions. The samples were embedded in Agar 100 resin and a microtome (Reichert Ultracut S Ultra microtome, Leica Biosystems, Nussloch, Germany) was used to cut the samples into 60 nm thin sections and stained with uranyl/lead. A transmission electron microscope (Jeol JEM-1230) at the UiB imaging core facility was used to acquire images.

### 2.4. Sample Preparation for TMT-Based Proteome Analysis

Muscle samples from 4 and 12-month-old DJ-1 KO and WT fish were snap-frozen in liquid nitrogen and stored at −80 °C until further use. Tissues were homogenised by sonication (3 × 30 s, 30% amplitude) in 4% Sodium dodecyl sulfate (SDS) in 100 mM Tris, pH 7.6, using 12 µL/mg sample, but not less than 150 µL. Thereafter, samples were boiled at 95 °C for 5 min and cleared by centrifugation (15,000× *g* for 15 min at 15 °C). Protein concentrations in supernatants were measured using the BCA assay.

In total, 25 µg of protein was reduced and alkylated in one step using a final concentration of 5 mM Tris (2-carboxyethyl) phosphine/10 mM Chloroacetamide/0.1 M Tris-HCl, pH 8.5. Furthermore, the protein clean-up and trypsin digestion were performed on Sera-Mag speed beads (cat. No 45152105050250 and 65152105050250, Cytiva, Marlborough, MA, USA) [16]. Two additional washes were added to ensure the complete removal of SDS from the samples before adding 0.8 µg of trypsin (Promega, V5111, Fitchburg, WI, USA) in 100 mM ammonium bicarbonate/1 mM CaCl_2_. The samples were sonicated for 30 sec in a water bath before incubation at 37 °C overnight.

After digestion was completed, the tubes were placed on the magnet, and the supernatant was removed and collected in a new tube before 50 µL of 0.5 M NaCl was added to the samples. This was followed by sonicated for 30 sec in a water bath. The tubes were placed on the magnet and the supernatant transferred to the corresponding tube. The peptides were acidified with 10% trifluoroacetic acid (TFA) to a pH of 2–3, and further diluted with 0.1% TFA to a final volume of approximately 300 µL before desalting the samples on a pre-wetted OASIS C18 HLB 96-well plate (2 mg sorbent per well, 30 µm, part no 186001828BA). The samples were washed 3 times with 0.1% TFA and eluted twice with 70% acetonitrile (ACN) and 0.1% formic acid (FA). The eluted peptides were lyophilised and resuspended in 21.5 µL of 100 mM HEPES, pH 8.5, and the peptide concentration was measured using a NanoDrop. A total of 11 µg of each sample peptide was used for TMT labelling.

TMTpro16-plex isobaric labelling reagents (Thermo Fisher Scientific, Waltham, MA, USA, #A44521) were resuspended in 23 µL of anhydrous ACN, and each sample was labelled twice with 2.3 µL of TMT reagent. A total of 110 µg of TMT reagent was used per sample (10× the protein concentration). Labelling reaction was performed at 25 °C and 400 rpm for 30 min each. The total incubation time was 1 h. The TMT labelling reactions were quenched by adding 5% hydroxylamine in 50 mM HEPES, pH 8.5, up to a final concentration of 0.4%, for 15 min at 25 °C and 400 rpm. All 16 samples were combined and acidified with 10% TFA/10% ACN. The combined sample was divided into 100 µg aliquots and lyophilised.

The lyophilised sample was resuspended in 300 µL of 0.1% TFA, loaded onto a high-pH reversed-phase fractionation column (Thermo Fisher Scientific, Waltham, MA, USA, # 84868), and eluted in eight fractions according to the manufacturer’s instructions for TMT-labelled peptides. The fractions were lyophilised and resuspended in an appropriate volume of 0.1% FA for LC-MS analysis.

### 2.5. LC-MS Analysis of TMT-Labelled Samples

About 0.5 μg of protein in the form of tryptic peptides dissolved in 2% ACN and 0.5% FA were injected in an Ultimate 3000 RSLC system (Thermo Scientific, Sunnyvale, CA, USA) connected online to an Orbitrap Eclipse mass spectrometer (Thermo Scientific, Bremen, Germany) equipped with an EASY-spray nano-electrospray ion source (Thermo Scientific).

The sample was loaded and desalted on a pre-column (Thermo Scientific, Sunnyvale, CA, USA, Acclaim PepMap 100, 2 cm × 75 µm ID nanoViper column, packed with 3 µm C18 beads) at a flow rate of 5 µL/min for 5 min with 0.1% TFA.

Peptide separation was conducted as described in Chavali et al. [12], except the total LC-run time was 140 min.

Peptides eluted from the column were detected in the Orbitrap Eclipse Mass Spectrometer with FAIMS [17] enabled using two compensation voltages (CVs): −50 V and −70 V. During each CV, the mass spectrometer was operated in the DDA mode (data-dependent acquisition) to automatically switch between one full scan MS and MS/MS acquisition. Instrument control was through Orbitrap Eclipse Tune 3.5 and Xcalibur 4.5. The cycle time was maintained at 1.5 s/CV. MS spectra were acquired in the scan range 375–1500 *m*/*z*, with resolution R = 120,000 at *m*/*z* 200, automatic gain control (AGC) target of 4 × 10^5^, and the maximum injection time (IT) set to Auto. The most intense elution peptides with charge states 2 to 6 were sequentially isolated to a target value (AGC) of 1 × 10^5^ and a maximum IT of 120 ms in the C-trap. Isolation width was maintained at 0.7 *m*/*z* (quadrupole isolation) before fragmentation in the HCD (Higher-Energy Collision Dissociation). Fragmentation was performed with a normalised collision energy (NCE) of 32%, and fragments were detected in the Orbitrap at a resolution of 50,000 at *m*/*z* 200, with the first mass fixed at *m*/*z* 110. One MS/MS spectrum of a precursor mass was allowed before dynamic exclusion for 30 s with “exclude isotopes” on. Lock-mass internal calibration was not enabled. The spray and ion-source parameters were as follows: ion spray voltage = 2000 V, no sheath and auxiliary gas flow, and capillary temperature = 275 °C.

Data analysis was performed with Proteome Discoverer Version 2.5. The database file used for the SequestHT search was UniprotKB danio reiro (Uniprotkb_Danio_rerio_id_7955_47577entries_Apr2024.fasta). The following setting was used: 20 ppm precursor mass tolerance; 0.5 Da fragment mass tolerance; static modification: TMTpro/+304.207 Da (K); static modification: carbamidomethyl (C); and dynamic modification for methionine oxidation. The maximum of missed cleavage sites was set to 2, with a minimum peptide length of 6. The validation settings were 0.01 for strict PSM false discovery rate (FDR) using a target–decoy strategy and 0.05 for relaxed.

Proteins included for further analysis were selected based on having at least two unique peptides (Supplementary Table S1), their level of fold change, and *p*-value. Proteins with a fold change of ≥1.5 were classified as upregulated, and those with a fold change of 0.67≤ were classified as downregulated. *p*-values were calculated using an independent two-sample *t*-test to compare the mean of KO and WT samples at 4 and 12 months, with a significance level of 0.05 (Supplementary Tables S2 and S3).

### 2.6. Analysis of Proteomics Data

Annotation of proteins to their cellular compartment was conducted by combining information from the zfin database (2024-06-19 GAF format), UniProtKB (2024-09-16), and Gene Ontology (2024-06-17 OBO). The annotation process was conducted using the Python programming language (v3.10) with the free libraries pandas, numpy, scipy, goatools, seaborn, and matplotlib.

Regulated protein data were exported from UniProtKB (JSON format) using the id-mapper tool and selected by protein accessions. Proteins were assigned to cellular compartments by checking if their GO terms mapped onto any root terms associated with organelles using relationships in the GO database. This process was repeated with the zfin database instead of UniProtKB. The output data from both steps were merged, and duplicate entries for each organelle were removed.

The protein–protein interaction networks were established based on proteins that were considered regulated (Supplementary Tables S2 and S3), using cytoscape (V 3.10) with the STRING network plugin and the zebrafish genome as a background. Proteins without any interactions were removed from the network. To establish clusters of proteins with shared function, interacting proteins were further grouped using STRING and the MCL function with an inflation value of 4. The identified clusters were manually colour-highlighted, and their functions labelled.

### 2.7. Targeted UPLC-HRMS Metabolomic Analysis

Skeletal muscle tissue was sampled from 9–24-month-old male WT and DJ-1-knockout zebrafish. A 400 µL volume of ACN:/CH_3_OH:/H_2_O (50:40:10, *v*:*v*:*v*) and 6 zirconium oxide beads (2.8 mm) were added to each muscle sample. The tissue samples were homogenised using FastPrep-24 Classic at 10 m/s for 2 × 20 s, followed by sonication for 15 min in an Ultrasonic ice bath. The samples were vortexed for 3 × 5 s. The lysate samples were then centrifuged at −2 °C for 10 min at 16,200× *g*. Both supernatant and pellets were stored at −80 °C prior to analysis.

Targeted metabolomic analysis of the supernatant was conducted, while the pellet was used to detect the amount of protein in the sample. Ultra-performance liquid chromatography with high-resolution mass spectrometry (UPLC-HRMS) was conducted using a Thermo QExactive (HRMS) mass spectrometer interfaced with the Dionex UltiMate 3000 (UPLC) liquid chromatography system (Thermo Fisher Scientific, Sunnyvale, CA, USA). Heated electrospray ionisation (H-ESI) was used. Separation was performed on an Atlantis Premier BEH Z-HILIC VanGuard FIT Column (Waters, Selangor, Malaysia) using 30 mM ammonium carbonate in Milli-Q water at pH 7 as mobile phase A and acetonitrile as mobile phase B with a flow rate of 0.45 mL/min. The following gradient was used: 78% of B for 3 min, decreased to 60% over 18 min and subsequent 10 min of column equilibration with 78% of B. The column compartment was maintained at a temperature of 25 °C during analysis, and the total runtime was 30 min. An injection volume of 10 μL was used, and the autosampler temperature was set to 5 °C.

Ions were monitored in positive targeted single ion monitoring (t-SIM) mode with a resolution of 70,000 at *m*/*z* = 200 and an isolation window of 8 *m*/*z*. Sheath gas flow rate was set to 48 (arbitrary units), aux gas flow rate to 11 (arbitrary units), sweep gas flow rate to 2 (arbitrary units), spray voltage to 3.5 kV, capillary temperature to 256 °C, S-lens RF level to 30, AGC (automatic gain control) target to 2 × 10^5^, and maximum injection time to 200 s.

Exact mass acquisition and relative quantification of polar metabolites were carried out using the Thermo XCalibur Quan Browser software 4.0.27.42, with 5 ppm mass tolerance and an in-house library of chemical standards as a reference.

## 3. Results

### 3.1. DJ-1 Loss Induces Skeletal Muscle Atrophy and Sub-Sarcolemmal Mitochondrial Degeneration

**Figure 1:**
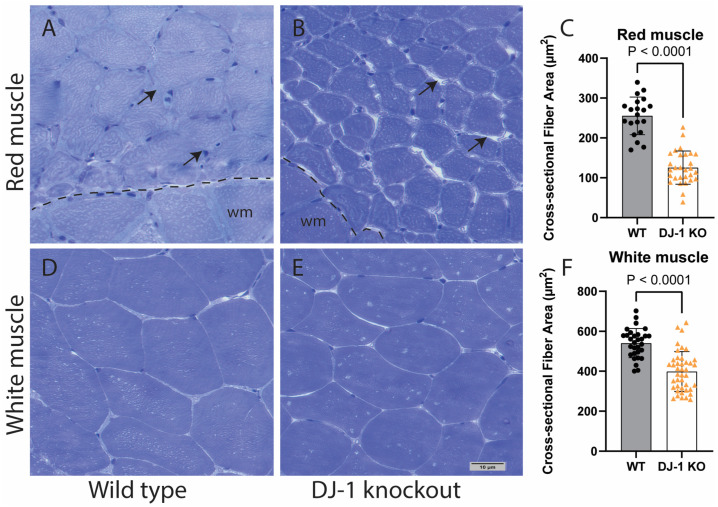
Loss of DJ-1 induces muscle atrophy in zebrafish. Semi-thin cross sections of red (**A**,**B**) and white (**D**,**E**) muscles from two-year-old male zebrafish were stained with Toluidine Blue (Scale bar 10 µm). Arrows point to extracellular space. White muscles appearing in A and B are labelled wm and separated from red muscle with dotted line. Cross sections were scanned and fibre cross-sectional area measured for red (**C**) and white (**F**) muscles. Data are the median ± sd (*n* = 29–42 DJ-KO and *n* = 20 WT). *p*-values were obtained using the Mann–Whitney U test.

We have previously shown that *park7*-knockout zebrafish have reduced swimming performance compared to wild-type animals at the 6-month stage [14]. Additionally, at the 9-month stage, there is a significant weight difference between knockout and wild-types, which further increases with ageing [14]. In fish, the red mitochondrial-dependent slow-twitch muscle is separated from the white glycolytic fast-twitch muscle, with the latter comprising the majority of the muscle mass. We expected the observed difference in weight between knockout and wild-type fish to be reflected in muscle mass. Indeed, cross-sections of muscle showed severe distinction in morphology (Figure 1 and Supplementary Figure S1). In particular, this was prominent in the red muscle, in which muscle fibres had a 2.2-fold reduction in cross-sectional area (Figure 1C). Red muscle also appeared to have increased extracellular space between myofibers (Figure 1A,B, arrows). Morphological changes in the white muscle were less prominent (Figure 1D,E), although a 1.4-fold reduction in cross-sectional area was observed (Figure 1F).

**Figure 2:**
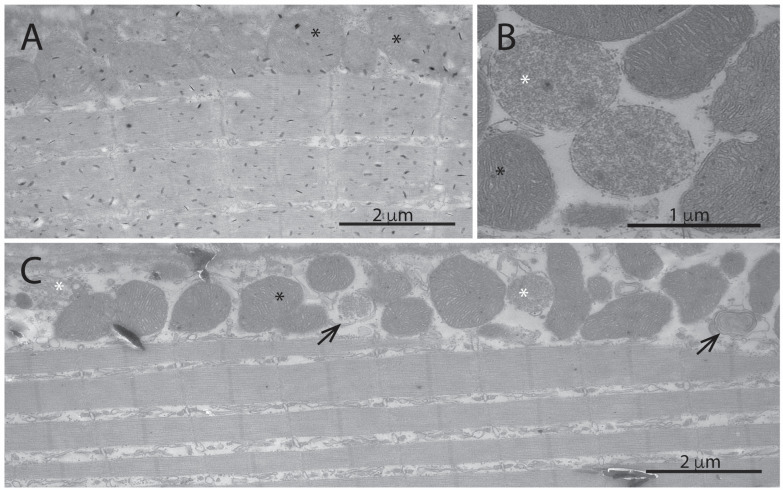
DJ-1-knockout animals show a high degree of degenerating sub-sarcolemma mitochondria in red muscle. Figure shows transmission electron micrographs of red muscle from 18-month-old wild-type (**A**) and DJ-1-knockout (**B**,**C**) zebrafish. Panel B shows examples of healthy (black asterisk) and degenerating (white asterisk) mitochondria found in red muscle. In wild-type red muscle, mitochondria appeared healthy and no degenerating mitochondria were observed in the sub-sarcolemma (**A**). In the DJ-1 knockout, a high degree of degenerating mitochondria and autophagic structures (arrows) in the sub-sarcolemma were observed (**C**).

DJ-1 has been shown to be important for mitochondrial function, in particular in response to oxidative stress [18]. We have previously shown that there is a marked reduction in mitochondrial complex I in the adult zebrafish DJ-1-knockout muscle [9]. Electron microscopy imaging of the highly mitochondrial-dependent red muscle revealed marked ultra morphological changes in the sub-sarcolemma area of the DJ-1 knockout (Figure 2B,C, asterisks) compared to the wild-type (Figure 2A, asterisks). In DJ-1 knockouts, disrupted morphology and inner structure of mitochondria were frequently seen together with autophagic structures (Figure 2B,C, arrows).

### 3.2. Proteomic Profiling of the Ageing Skeletal Muscle Proteome in Wild-Type and DJ-1 Knockout

Skeletal muscle from 4 and 12-month-old wild-type and DJ-1-knockout animals were sampled for TMT-based proteomics. Sampling points were chosen based on earlier obtained behaviour data in which knockout animals showed a significant decrease in mobility between 6 and 9 months of age [12]. Muscle tissues were resected from the trunk muscle posterior to the dorsal fin. Tissue samples included mainly white muscle, as well as red and intermediate muscle. In total, we identified 3721 proteins. Only proteins identified with a minimum of two unique peptides and identified in all samples were used for further quantitative analysis (Supplementary Table S1) (2184 proteins). No proteins were identified in only WT or vice versa. When comparing knockout and wild-type muscles, 68 proteins were found to be significantly (*p* > 0.05) regulated with a fold change of 1.5 or more both at 4 months (Supplementary Table S2) and 12 months (Supplementary Table S3).

**Figure 3:**
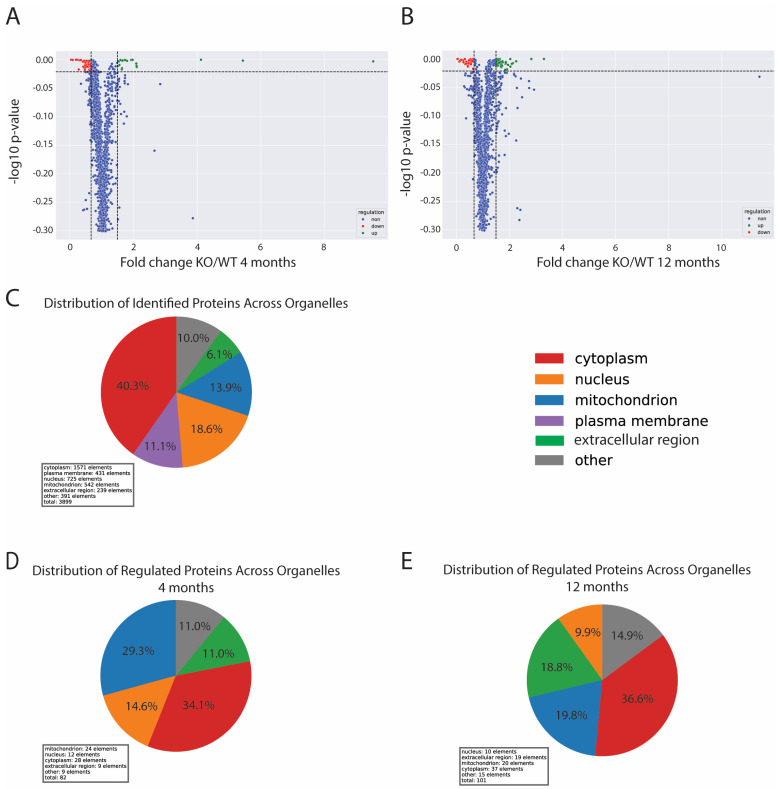
Changes in the muscle proteomes of early and late adults. Figure shows volcano plots of the distribution changes in 4-month-old (**A**) and 12-month-old (**B**) DJ-1-knockout zebrafish muscles compared to their respective wild-types. Proteins with significant (*p* < 0.05) changes larger than 1.5-fold are indicated in green (upregulated) and red (downregulated). Proteins not regulated are in blue. (**C**) Cellular distribution of total identified proteins used for quantitation. Cellular distribution of regulated proteins when comparing DJ-1-knockout muscles and wild-type muscles at 4 (**D**) and 12 (**E**) months.

The most striking changes when comparing DJ-1 knockout and wild-type animal muscle proteomes were the altered levels of proteins associated with mitochondria and the extracellular region (Figure 3). In the young knockout animals, mitochondrial proteins comprised a large part (29.3%) of the regulated proteins, whilst regulated extracellular proteins accounted for 11%. In the late knockout adults, regulated mitochondrial proteins (19.8%) were still prominent. At this late stage, the number of regulated proteins belonging to the extracellular region (18.8%) was further increased, thus reflecting an increased inflammatory response.

**Figure 4:**
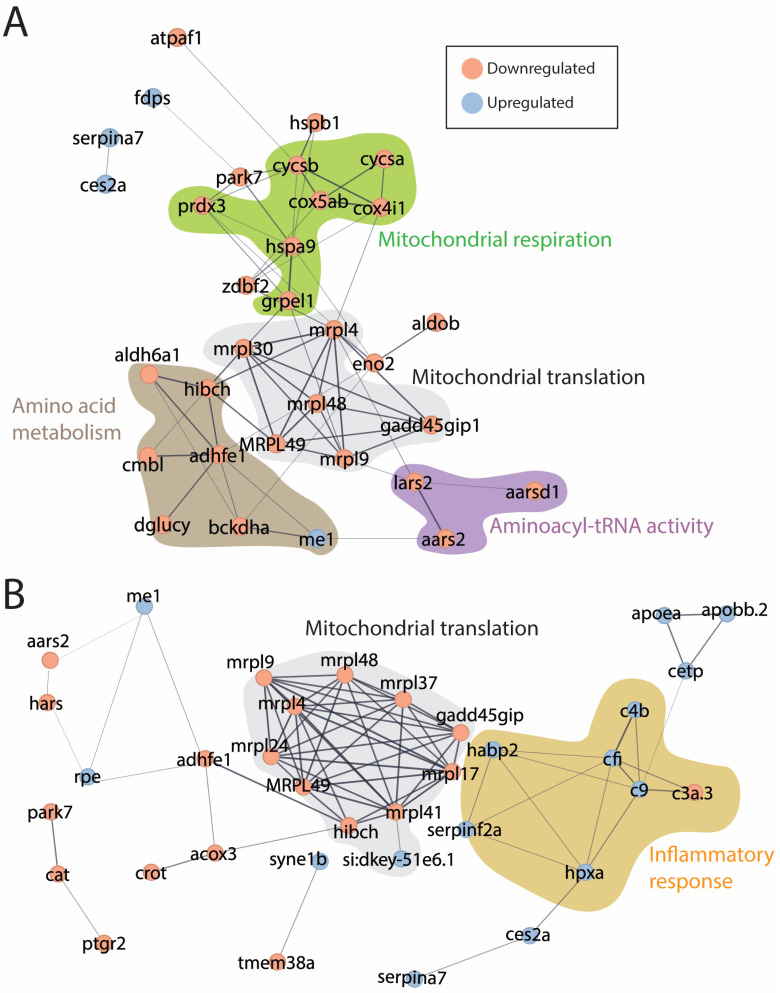
Interaction networks of regulated proteins. (**A**) 4 months and (**B**) 12 months. The interaction networks were established from regulated proteins (Supplementary Tables S2 and S3) using Cytoscape (V 3.10) with the STRING network plugin and the zebrafish genome as a background. Proteins without any known interactions were removed.

A Cytoscape/STRING network analysis identified functional categories of early regulated proteins, including mitochondrial respiration/translation, amino acid metabolism, and aminoacyl-tRNA activity (Figure 4A). In the 12-month-old muscle, the functional clusters of regulated proteins belonged to mitochondrial translation and inflammatory responses (Figure 4B).

Table 1 gives an overview of the proteins with 2-fold or more regulation in skeletal muscles in 4 and 12-month-old animals. In these young adults, motor dysfunction and loss of weight gain have not yet appeared [12]. Among the most regulated proteins, proteins associated with either inflammatory control or mitochondrial function and metabolism still constituted the majority.

All 2-fold or more regulated mitochondrial-associated proteins were downregulated. Their function could be grouped into metabolism (Alcohol dehydrogenase iron-containing 1, 2,4-dienoyl CoA reductase 1, 3-hydroxyisobutyryl-CoA hydrolase), oxidative phosphorylation (Cytochrome c oxidase subunit 5Ab), mitochondrial gene expression (Mitochondrial ribosomal protein L49, 4, and 30, Large ribosomal subunit protein mL64, Alanyl-tRNA synthetase 2), and mitochondrial-specific protein dephosphorylation (Protein phosphatase, Mg^2+^/Mn^2+^ dep.Ka). Among the mitochondrial-associated proteins, 2,4-dienoyl CoA reductase 1 (DECR1) was the most dysregulated. DECR1 is necessary for oxidation of unsaturated fatty acids and adaption to mitochondrial stress [19].

Annexin A3 and Acid sphingomyelinase-like phosphodiesterase were the two most upregulated proteins in young adults (Table 1). Both proteins are associated with early inflammatory responses [20,21]. Annexin A3 and another highly upregulated protein, glutamate receptor, have been linked to motor nerve injury responses [20,22]. Both Acid sphingomyelinase-like phosphodiesterase and glutamate receptors were still upregulated in the 12-month-old adult muscle, but increased Annexin A3 expression appeared transient (Table 1 and Supplementary Table S3).

The most downregulated proteins in both early and old adults were actinin alpha a/b, two major components of the Z-disc through which mutations can cause skeletal myopathies (Table 1 and Supplementary Table S3) [23].

The most prominent difference between young and late-adult animal groups was the marked change in Cytosolic glycerol-3-phosphate dehydrogenase (*gpd1b*) levels. This NAD-dependent enzyme has an important link between carbohydrate and fat metabolism [24]. In the early adults, no difference was observed when comparing knockout and wild-type animals. In the late adults, however, cGPD levels were 20-fold higher in wild-types (Supplementary Tables S2 and S3).

Most proteins that were highly downregulated in late-stage DJ-1-knockout muscle were also observed to be downregulated at an earlier stage (Supplementary Tables S1–S3), with one exception: Prostaglandin reductase 2.

### 3.3. DJ-1 Knockout Accelerates Age-Dependent Decrease in Skeletal Muscle NAD^+^/NADH Ratio

**Figure 5:**
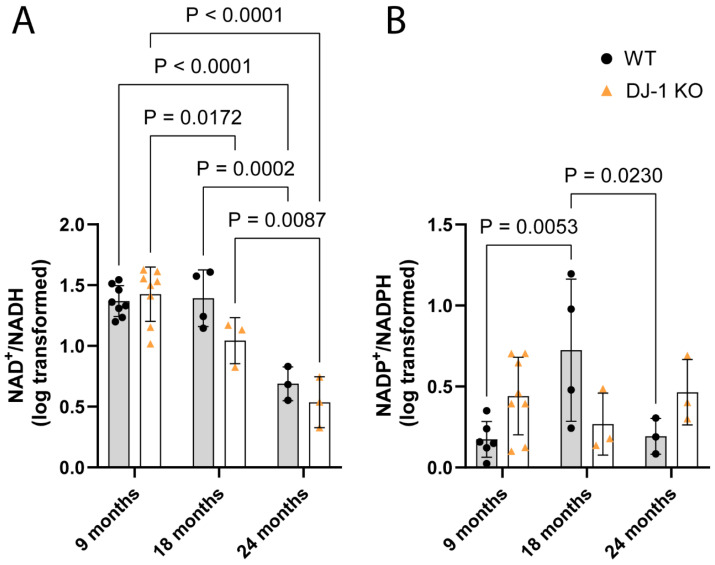
Absence of DJ-1 facilitates an age-dependent decrease in muscle NAD^+^/NADH ratio. Metabolites were measured using targeted mass spectrometry on whole muscle from different age groups. A two-way ANOVA analysis was conducted on the (**A**) NAD^+^/NADH and (**B**) NADP^+^/NADPH ratios of 10 log-transformed data. A significant age-dependent reduction in the NAD^+^/NADH ratio was found in both DJ-1 KO and WT (F (2, 23) = 37.78, *p* < 0.0001). No significant age-dependent effect was found in the NADP^+^/NADPH ratio (F (1, 21) = 0.007642, *p* = 0.2532). The represented *p*-values comparing age groups from the same genotype were obtained from a Tukey multiple comparisons test on the Log10-transformed data.

Ageing human skeletal muscles show a decline in NAD^+^ levels [25] and a lowering of the NAD^+^/NADH ratio [26]. A significant age-dependent reduction in the NAD^+^/NADH ratio was also observed in both wild-type and *park7* knockouts. In the wild-types, an age-dependent decline in the NAD^+^/NADH ratio occurred between 18 and 24 months of age (Figure 5A). In *park7* knockouts, however, this decline was observed between 9 and 18 months of age (Figure 5A). NAD^+^ is a precursor for the synthesis of NADP, which in its reduced form, NADPH, acts as an electron donor to antioxidants, e.g., glutathione [27]. With an age-dependent reduction in NAD^+^ availability and an expected increase in oxidative stress in the knockout, one would expect the NADP^+^/NADPH ratio to be affected. However, no significant age-dependent change in the NADP^+^/NADPH ratio was observed in the *park7* knockouts (Figure 5B). On the other hand, the NADP^+^/NADPH ratio was significantly higher in the 18-month-old wild-types compared to both the 9 and 24-month-old wild-type animals. At this stage, no change in the NAD^+^/NADH ratio had appeared. This result was rather unexpected and might be influenced by a low number of animals, as the variation between samples at this 18-month stage was large.

## 4. Discussion

DJ-1 is a well-established defender against oxidative stress and mitochondrial dysfunction [3,28]. DJ-1 levels decrease in ageing human skeletal muscle [4], thus reducing the potential to counteract (ageing-mediated) age-dependent increase in oxidative stress [29].

Here, we showed that loss of DJ-1 in the zebrafish induces skeletal muscle atrophy and mitochondrial degeneration. We identified dysregulations in the skeletal muscle proteome of early adult DJ-1-knockout zebrafish that occur at a stage prior to the appearance of motor symptoms and absence of weight gain [12].

DJ-1-knockout zebrafish show dysfunctional swimming behaviour and loss of weight gain between 6 and 9 months of age [12]. This seems to be a result of muscle atrophy, in which the slow mitochondria-dependent red skeletal muscle seems to be particularly affected (Figure 1). Degeneration of the sub-sarcolemma mitochondria in the knockout red muscle is frequently observed (Figure 2). In contrast, the interfibrillar mitochondria seem less affected. This may be a result of uneven oxidative stress exposure as ageing skeletal muscle sub-sarcolemma mitochondria are known to produce higher amounts of reactive oxygen species and increased degradation compared to the interfibrillar subtype [30].

To search for cellular changes responsible for the reduction in muscle mass and function, we performed a quantitative proteomics analysis in young adults (4 months) at a timepoint preceding dysregulation in swimming behaviour and loss of weight gain. At this stage, only approx. 3% of the identified skeletal muscle proteins were found to have an expression level change of 1.5-fold or more when comparing DJ-1 knockout and wild-type animals. Among these, proteins associated with the mitochondria and extracellular space were most pronounced, thereby indicating both early existing mitochondrial dysfunction and inflammatory response (Figure 3).

One might expect that the lack of antioxidant function of DJ-1 would be compensated for with increased expression of other antioxidant proteins; however, this is not the case. Antioxidant proteins like SOD1, SOD2, and catalase showed no increase induced by the loss of DJ-1; on the contrary, a small decrease in their expression levels was observed (Supplementary Table S1). This supports previous work that DJ-1 may act upstream of NRF2, which regulates the transcription of these antioxidant response proteins [9,31]. Reduced antioxidant capacity would, therefore, increase oxidative stress and presumably lead to mitochondrial dysfunction. We have previously shown that loss of DJ-1 in zebrafish results in downregulation of mitochondrial complex I activity in skeletal muscle [9]. Also, mitochondrial complex I was affected in a rodent model with muscle-specific DJ-1 knockdown [4]. Zhang et al., like us, also found DJ-1 knockout to affect mitochondrial ultrastructure and reduce muscle fibre size, although the effect on fibre size was less than what we observed in the zebrafish model (Figure 1 and Figure 2). In contrast to zebrafish DJ-1 knockout, the rodent model did not show any change in the expression of genes related to mitochondrial oxidative phosphorylation. In the zebrafish skeletal muscle, loss of DJ-1 reduced the protein levels of a number of mitochondrial proteins with functions associated with mitochondrial gene transcription, oxidative phosphorylation, and metabolism, both in young and old adults (Table 1, Figure 4, and Supplementary Tables S2 and S3).

DJ-1 regulates stress responses and survival by activating the relevant pathways by inhibiting phosphatase PTEN [32] and thereby increasing Akt phosphorylation [33]. The effect of the loss of DJ-1 in rodent muscle was suggested to be the result of decreased Akt phosphorylation inducing translocation of the transcription factor FoxO1 [4].

The most upregulated protein in the early adult zebrafish knockout muscle was Annexin A3 (Table 1). Annexin A3 is involved in pro-inflammatory responses and angiogenesis [34]. This upregulation was transient in the DJ-1 knockout, in contrast to most other regulated proteins associated with inflammation (Table 1 and Supplementary Table S3). Annexin A3 is also highly expressed in myocardial infarction, and silencing Annexin A3 promotes myocardial repair after acute ischemic injury through activation of the PI3K/AKT pathway [35]. Thus, both the presence of DJ-1 and the downregulation of Annexin A3 may act as stress protection. The absence of DJ-1 and the increased expression of Annexin A3 may reduce the ability to activate the pro-survival PI3K/AKT pathway. A similar effect has been suggested to be the result of increased Acid sphingomyelinase-like phosphodiesterase 3b expression [36], which we also observe in the DJ-1-knockout muscle (Table 1 and Supplementary Table S3).

In early DJ-1-knockout adults, Actinin 2 was the most downregulated protein (Table 1). Actinin 2 crosslinks actin fibres and is an essential component of the muscular Z-disc. Mutations or frameshifts in *ACTININ2* cause myopathy both in cardiac and skeletal human muscle [23]. A crosstalk between Actinin 2 regulation and mitochondrial dysfunction has been linked to a reduction in the NAD^+^/NADH ratio and cardiomyopathy [37].

The most downregulated mitochondrial-associated protein was 2,4-dienoyl CoA reductase 1 (DECR1). DECR1 has an important function in mitochondrial homeostasis, and its downregulation increases oxidative stress [38]. Increasing DECR1 levels after myocardial infarction ameliorates mitochondrial structural damage and bioenergetic dysfunction [39].

As in humans, zebrafish show age-related loss of skeletal muscle mass and function, termed sarcopenia [40]. Here, we showed that the loss of DJ-1 not only accelerates the main morphological features of sarcopenia but also affects proposed molecular hallmarks of ageing.

An age-dependent feature observed in various tissues, including skeletal muscle, is a decline in NAD and an increase in the NAD^+^/NADH ratio [41,42]. The co-enzyme NAD is highly integrated in cellular metabolism, and the maintenance of NAD^+^/NADH balance is dependent on mitochondrial Complex I activity. Also, a decline in NAD^+^/NADH was observed in the ageing zebrafish skeletal muscle (Figure 5). In the DJ-1 knockout, however, this decline occurred at an earlier stage compared to the wild-type.

Reductions in Prostaglandin reductase 2 and Glycerol-3-phosphate dehydrogenase levels have been proposed as hallmarks of ageing tissues [43,44,45]. The levels of both of these enzymes were lower in the DJ-1-knockout muscle compared to the wild-type (Supplementary Table S3). Additionally, the DJ-1-knockout model has a reduced level of Complement C3 (Table 1 and Supplementary Table S3), another biomarker associated with sarcopenia [46].

In summary, loss of DJ-1 leads to mitochondrial dysregulation and sustained inflammation, which eventually results in skeletal muscle atrophy. We showed that the absence of DJ-1 in zebrafish accelerates both morphological and proposed molecular hallmarks of human sarcopenia.

## Figures and Tables

**Table 1:** Proteins regulated more than two-fold in skeletal muscle from 4 and 12 months old animals.

			Peptides		Score	KO/WT			Cell Compart-ment	
Acc.no	Gene	Protein Name	Total	Unique		4 Mnts	12 Mnths	*p*-Value		Biological-/Molecular Function
**Inflammation related**										
A8E5E5	*anxa3b*	Annexin	8	8	25	9.5		**		
A0A8M1P530	*smpdl3b*	Sphingomyelin phosphodiesterase acid like 3B	2	2	8	5.4	3.3	**	ex.c	sphingomyelin catabolic process
A0A8M1Q7M7	*emilin2a*	Elastin microfibril interfacer 2a	2	2	8	2.1		*	ex.c	
Q6NYX8	*pithd1*	PITH domain containing 1	2	2	6	2.1		*	n, c	positive regulation of megakaryocyte differentiation
A0A8M9Q4F0	*LOC110439158*	Alpha-1-antiproteinase 2-like	7	4	28	2.1		*	ex.c	serine-type endopeptidase inhibitor activity
F1QX13	*c3a.3*	Complement C3a	21	3	76	0.2	0.3	***	ex.c	immune response
Q7ZUH8	*mgst3a*	Microsomal glutathione S-transferase 3a	2	2	12	0.3		*	n,er,m	leukotriene metabolic process
Q804G3	*anxa11b*	Annexin A11b	4	2	24	0.5		*	pm, c	phagocytosis, cytokinetic process
**Mitochondrial translation and metabolism**										
A0A8M1N0E2	*decr1*	2,4-dienoyl CoA reductase 1	5	5	25	0.2	0.2	***	m	fatty acid beta-oxidation
Q6NYM8	*adhfe1*	Alcohol dehydrogenase iron containing 1	2	2	7	0.4	0.3	**	m	alcohol dehydrogenase (NAD+) activity
A0A8N7T6Q4	*mrpl49*	Mitochondrial ribosomal protein L49	2	2	6	0.4	0.2	*	m	translation
A0A0R4ILL2	*mrpl4*	Mitochondrial ribosomal protein L4	2	2	4	0.5	0.2	*	m	translation
Q502J9	*mrpl30*	Mitochondrial ribosomal protein L30	2	2	5	0.5		*	m	translation
A0A8M1Q6V5	*aars2*	Alanyl-tRNA synthetase 2	3	3	11	0.5		*	m	translation
A0A8M1PVC0	*gadd45gip1*	Large ribosomal subunit protein mL64	2	2	5	0.5		*	m, n	cell cycle
Q58EB4	*hibch*	3-hydroxyisobutyryl-CoA hydrolase	4	4	16	0.5		*	m	valine catabolic process
E7FAZ1	*ppm1ka*	Protein phosphatase, Mg^2+^/Mn^2+^ dep.Ka	2	2	7	0.5		**	m	peptidyl-threonine dephosphorylation
A0A0R4IVC8	*cox5ab*	Cytochrome c oxidase subunit 5Ab	5	2	32	0.5		*	m	mitochondrial electron transport
**Actin organization**										
A0A8M3AZ01	*actn2a*	Actinin, alpha 2a	3	2	28	0.1	0.1	***		
Q2YDR5	*actn2b*	Actinin, alpha 2b	14	3	169	0.1	0.1	***	pm	actin cytoskeleton organization, sarcomere organization
**Other**										
B8JLR6	*si:ch211-251b21.1*	Glutamate receptor	4	4	36	4.1		****	pm	synaptic transmission
Q4QRF7	*leg1b*	Protein leg1b	6	4	27	0.3		**	ex.c	
A0A8M2B601	*ampd3b*	AMP deaminase	7	3	12	0.4		**	c	AMP metabolic process
Q7T1E0	*gpd1b*	Glycerol-3-phosphate dehydrogenase	6	4	23		0.1	****	c	NADH oxidation
Q6DEI9	*ptgr2*	Prostaglandin reductase 2	2	2	64		0.3	*	c	Prostaglandin metabolic process
Q503F8	*crot*	Peroxisomal carnitine O-octanoyltransferase	2	2	6		0.3	****	p	Lipid metabolism

*p*-values: * < 0.05, ** < 0.01, *** < 0.001, **** < 0.0001; Cell compartment: extra cellular (ex.c), mitochondria (m), nucleus (n), cytoplasma (c), plasma membrane (pm), peroxisome (p).

## Data Availability

Proteomics data are available at https://dataverse.no/dataverse/uib (12 December 2024).

## Supplementary Materials

The following supporting information can be downloaded at: https://www.mdpi.com/article/10.3390/antiox13121509/s1, Supplementary Figure S1: Overview of images used in Figure 1 showing separation between red and white muscle types, Supplementary Table S1: Total identified proteins used for quantitative analysis from DJ-1-knockout and wild-type zebrafish skeletal muscle, Supplementary Table S2: Protein regulated 1.5-fold or more at the 4-month stage in DJ-1 knockout vs. wild-type skeletal muscle, Supplementary Table S3: Protein regulated 1.5-fold or more at the 12-month stage in DJ-1 knockout vs. wild-type skeletal muscle.
