# Supplementary figures and images for: Accelerated Sarcopenia Phenotype in the DJ-1/Park7-Knockout Zebrafish
Source: Antioxidants (Basel). 2024 Dec 11;13(12):1509. doi: 10.3390/antiox13121509 (PMC11673048; doi:10.3390/antiox13121509)

Wild type

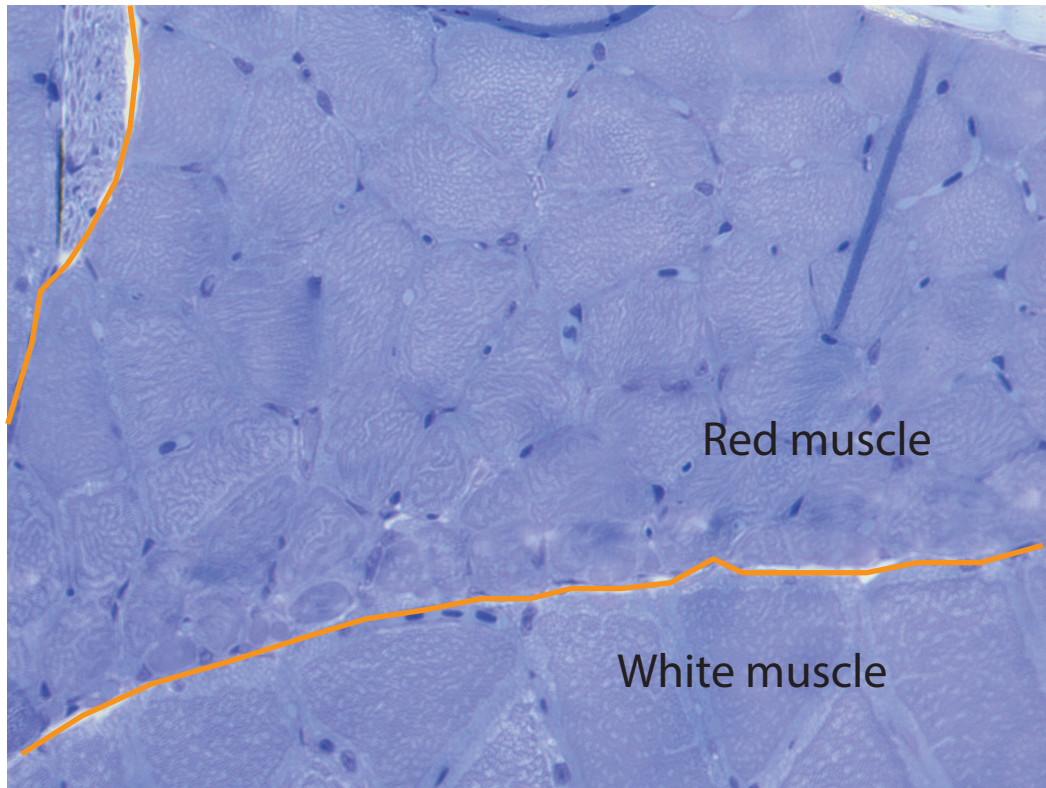

DJ-1 knockout

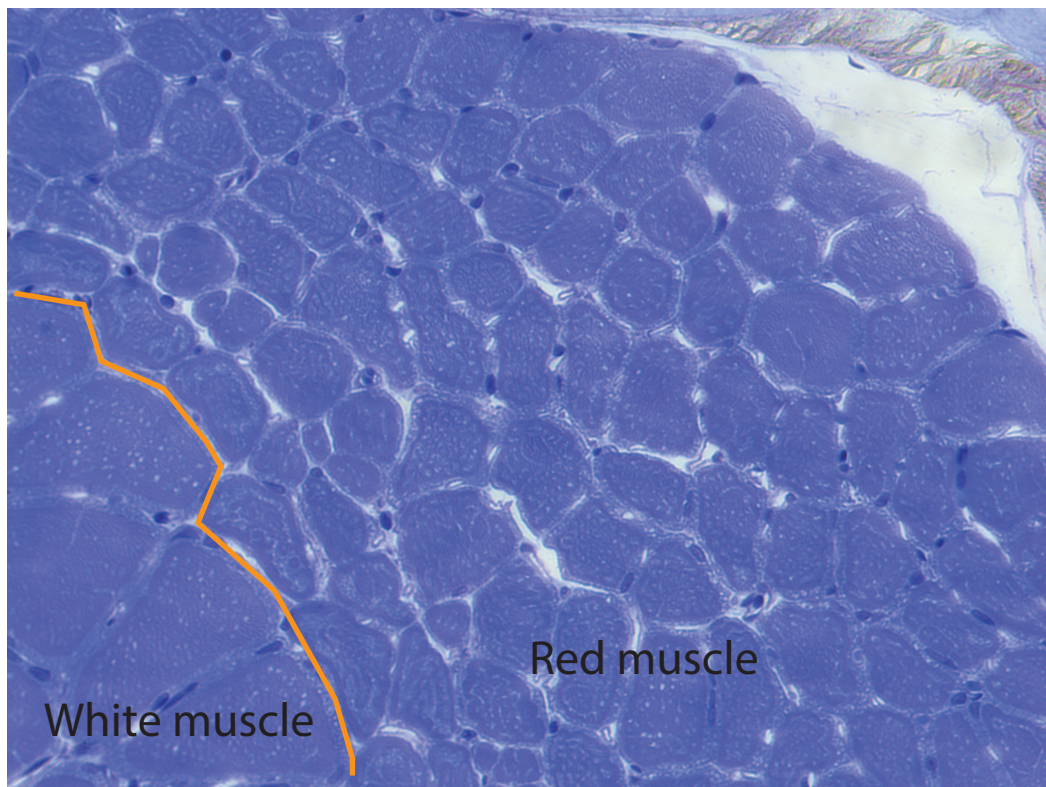

Supplement: Supplementary file 1 [file antioxidants-13-01509-s001.zip › Supp_Fig1.pdf]
